# Characterization of the HCMV-Specific CD4 T Cell Responses that Are Associated with Protective Immunity

**DOI:** 10.3390/v7082828

**Published:** 2015-08-06

**Authors:** Marie Wunsch, Wenji Zhang, Jodi Hanson, Richard Caspell, Alexey Y. Karulin, Mascha S. Recks, Stefanie Kuerten, Srividya Sundararaman, Paul V. Lehmann

**Affiliations:** 1 Department of Anatomy and Cell Biology, University of Wuerzburg, Koellikerstr 6, 97070 Wuerzburg, Germany; E-Mails: marie.wunsch@uni-wuerzburg.de (M.W.); stefanie.kuerten@uni-wuerzburg.de (S.K.); 2 Cellular Technology Limited, 20521 Chagrin Blvd, Shaker Heights, OH 44022, USA; E-Mails: wenji.zhang@immunospot.com (W.Z.); jodi.hanson@immunospot.com (J.H.); richard.caspell@immunospot.com (R.C.); ayk@immunospot.com (A.Y.K.); pvl@immunospot.com (P.V.L.); 3 Department of Anatomy I, University of Cologne, Joseph-Stelzmann-Str. 9, 50931 Cologne, Germany; E-Mail: mascha.recks@uk-koeln.de

**Keywords:** Human cytomegalovirus (HCMV), Enzyme-Linked Immunospot assay (ELISPOT), CD4 T cells, cytokine secretion, kinetics

## Abstract

Most humans become infected with human cytomegalovirus (HCMV). Typically, the immune system controls the infection, but the virus persists and can reactivate in states of immunodeficiency. While substantial information is available on the contribution of CD8 T cells and antibodies to anti-HCMV immunity, studies of the T_H_1, T_H_2, and T_H_17 subsets have been limited by the low frequency of HCMV-specific CD4 T cells in peripheral blood mononuclear cell (PBMC). Using the enzyme-linked Immunospot^®^ assay (ELISPOT) that excels in low frequency measurements, we have established these in a sizable cohort of healthy HCMV controllers. Cytokine recall responses were seen in all seropositive donors. Specifically, interferon (IFN)-γ and/or interleukin (IL)-17 were seen in isolation or with IL-4 in all test subjects. IL-4 recall did not occur in isolation. While the ratios of T_H_1, T_H_2, and T_H_17 cells exhibited substantial variations between different individuals these ratios and the frequencies were relatively stable when tested in samples drawn up to five years apart. IFN-γ and IL-2 co-expressing polyfunctional cells were seen in most subjects. Around half of the HCMV-specific CD4 cells were in a reversible state of exhaustion. The data provided here established the T_H_1, T_H_2, and T_H_17 characteristic of the CD4 cells that convey immune protection for successful immune surveillance against which reactivity can be compared when the immune surveillance of HCMV fails.

## 1. Introduction

Asymptomatic infections by human cytomegalovirus (HCMV), a β-herpesvirus, are common with a prevalence between 60% and 100% in industrial and developing countries, respectively [1]. Various cell types including neuronal-, endothelial-, epithelial-, smooth muscle cells, and leucocytes can be infected during an acute phase of HCMV infection [2]. The virus is able to persist during the entire lifetime of the infected host, mostly in a dormant state. Periodically, however, the virus can reactivate with the viral replication cycle initiated and viral antigens exposed to lymphocytes. Activation and expansion of HCMV-specific lymphocytes will then force the virus back into its dormant state [3]. While cycles of dormancy and reactivation are characteristic for HCMV infections, little is known about the frequency of these cycles and the timing of reactivations since they mostly occur subclinically and go unobserved. Methods available for monitoring the reactivation are insufficient [4]. For example, HCMV detection in the blood by polymerase chain reaction (PCR) is only possible during pronounced viremia and has a low sensitivity [5]. Measuring HCMV-specific serum antibodies does not distinguish between the infected state versus reactivation. Recently it has been shown that monitoring Perforin and Granzyme B, both secreted by recently activated CD8^+^ effector T cells but not resting memory T cells, can permit the distinction between dormant and active HCMV infection [6]. These episodes of virus reactivations lead to recurring stimulations and expansions of HCMV-specific T cells as a result of which up to 10% of all memory T cells can be HCMV specific in healthy individuals [7]. However, these multiple T cell stimulations, due to recurring viral reactivations, can also lead to exhaustion and functional impairment of CD8^+^ T cells, characterized by a progressive decline in their ability to produce cytokines like IFN-γ and IL-2 [8,9,10,11]. Little is known about the stimulation effects of chronic antigen on HCMV-specific CD4 cells, their T_H_1, T_H_2, and T_H_17 lineage development, and possible exhaustion. 

The importance of immune control of HCMV replication is evident with immunocompromised individuals including transplant recipients, neonates, the elderly, cancer, and HIV patients, in whom uncontrolled HCMV replication can cause high mortality [3]. In addition, a correlation has been established between reactivation of HCMV infections and several autoimmune diseases, suggesting a contribution of HCMV-reactive T cells to target organ damage [12,13,14]. Hence, there is a need to understand cellular and humoral immunity in states in which HCMV is effectively controlled, as opposed to stating that the immune system fails to provide adequate protection. 

CD8 cells are important for controlling viral infections, in general, and also for HCMV, in particular [15,16,17,18], and this arm of immunity is rather well explored, also because high frequencies of HCMV-specific CD8+ cells facilitate such studies. In addition, there is evidence that antibodies and CD4 T cells are also important for controlling HCMV replication and preventing related diseases [19,20] being in line with what is known for most viral infections. For example, in renal transplant recipients suffering from HCMV-induced disease, the HCMV-specific memory CD4 T cell response was found to be diminished compared to asymptomatic infections [21] and in stem cell transplant recipients an increased level of IFN-γ secreting HCMV-specific CD4 T cells was associated with a drop of virus load [22].

CD4+ T-cell-mediated defense relies on engaging independent CD4 cell lineages that mediate their activity by secreting certain cytokines in a somewhat exclusive manner. T_H_1 cells directly inhibit viral replication by secreting IFN-γ [21]. T_H_2 cells secrete IL-4 thereby functioning as helper cells for B cell response and immunoglobulin class/subclass switching [23]. An IL-17 producing CD4 subset, T_H_17 cells, mediates delayed type hypersensitivity (DTH) recruiting and activating macrophages at the site of antigen encounter for host defense [24]. The control of infections critically relies on engaging (or avoiding the engagement of) the T_H_1, T_H_2, and T_H_17 subsets which occurs through instructed differentiation induced by the antigen encounter. For HCMV, the extent of T_H_1, T_H_2, and T_H_17 subsets that are engaged in controlling the infection in clinically healthy individuals (“HCMV controllers”) is largely unknown. Moreover, the mechanism by which CD4 cell lineages contribute to the failure of immune surveillance in episodes of HCMV reactivations is unidentified. This is partly due to the fact that HCMV-specific CD4 cells occur in low frequencies for detection by flow cytometry analysis [25]. However, understanding these CD4 subsets in states of health may provide a framework and reference value for understanding these CD4 subsets during the failure of HCMV immune surveillance. The primary scope of this study is to establish the presence and relative ratios of HCMV-specific T_H_1, T_H_2, and T_H_17 subsets in healthy HCMV controllers using a highly sensitive ELISPOT technology.

For the present study we took advantage of a PBMC library of 45 healthy donors permitting a cross-sectional sampling of the human population. Of these donors, six were available for longitudinal testing over seven months to five years, allowing us to address the stability of the HCMV-specific CD4 cell repertoire possibly also detecting subclinical-activation induced changes. All the healthy donors that we tested were infected with HCMV, as verified by the presence of serum antibodies, yet were clinically healthy. In such individuals, therefore, the CD4 cell effector functions engaged were suitable to control the infection; termed HCMV controllers.

We used UV-inactivated entire virions to recall CD4 cells because, in this way, all possible epitopes of the virus become amenable for antigen-processing and presentation, and, unlike the peptides commonly used for CD8 cell activation, the assay system becomes independent of the HLA-type of the test person. We will show that such antigens stimulate CD4 cells exclusively. The ELISPOT assays performed permits the detection of individual antigen-specific T cells that secrete cytokines. HCMV-antigen-induced IFN-γ production was measured to establish the frequency of T_H_1 cells within the PBMC at single cell resolution. Also CD4 cells that co-express IFN-γ and IL-2 were measured using an IFN-γ /IL2 double color assay, because these cells, a subset of T_H_1 cells, have been associated with protection in several infectious diseases [26,27,28]. IL-4 and IL-17 ELISPOT assays were performed to establish the frequencies of HCMV-specific T_H_2 and T_H_17 cells, respectively. Since it has been shown that IL-7 can restore cytokine production in exhausted T cells [8,10,29], we tested the PBMC in media that either was, or was not supplemented with IL-7. By establishing the types of CD4 cell subclasses, and the frequencies of each, we hope to provide reference values for healthy donors, against which uncontrolled HCMV infections can be compared.

## 2. Materials and Methods

### 2.1. Thawing and Handling of Cryopreserved PBMC

Cryopreserved Peripheral Blood Mononuclear Cells (PBMC) from 45 healthy human donors was selected from a reference database at Cellular Technology Ltd. (www.epbmc.immunospot.com CTL, Cleveland, OH, USA). All donors are HLA-typed and pre-characterized for T-cell activity. PBMC were thawed according to an optimized protocol for optimal recovery and functionality of cryopreserved PBMC [30]. Briefly, cryovials were stored in nitrogen vapor until being placed in a 37°C glass bead bath for 10 min (CTL-BB-001, CTL). The cells were diluted slowly with pre-warmed 37 °C Anti-Aggregate medium from CTL (Cat # CTL-AA-005, CTL). PBMC were then centrifuged for 10 min, the supernatant was decanted, the cell pellet was tapped to re-suspend, and then Anti-Aggregate medium was added. A sample of the cells was then counted to determine viability using the Live/Dead/Apoptotic cell counting platform by CTL (Cat# CTL-LDAC-100, CTL). Once counted, cells were centrifuged again and then re-suspended at a final concentration of 2.5 × 10^6^ PBMC/mL of CTL-Test Medium (Cat# CTLT-005, CTL) in order to plate 100 μL (250,000 human PBMC) for each assay.

### 2.2. Antigen

UV-inactivated lysate of HCMV grade 2 was acquired from Cellular Technology Ltd. (Cat # CEF32-07-005, CTL). The HCMV lysate was dissolved at 100 μg/mL in CTL-Test Medium of which 100 μL was plated per well, resulting in a final concentration of 50 μL/mL. In experiments that aimed at establishing the dose response curve, the HCMV antigen was plated in serial dilution, as specified.

### 2.3. ELISPOT Assays

The 96-well ELISPOT assays were performed using human interferon-γ ImmunoSpot^®^ kit (CTL-HIFNG-1/5M), IL-2 ImmunoSpot^®^ kit (CTL-HIL2-1/5), IL-4 ImmunoSpot^®^ kit (HIL4-1/5) and IL-17 ImmunoSpot^®^ kit (CTL-HIL17-1/5) as well as the IFN-γ /IL-2 double-color enzymatic kit (CTL-HIFNGIL2-1/5) from Cellular Technology Ltd. Plates and all antibodies, tertiaries, diluents, and substrate are contained in the kits. The assays were performed according to the manufacturer’s instructions. Briefly, the PVDF membranes were pre-wet with 70% ethanol and the plates were coated with Human IFN-γ/IL-2/IL-4/IL-17 Coating Antibodies overnight. The next day, plates were washed once with PBS and then HCMV antigen was plated in CTL-Test Medium (unlike in Intracytokine staining (ICS), no signal enhancing antibodies were used in the assay). After plating the PBMC in 100 µL for the 96-well plate the plates were placed in a humidified incubator at 37 °C with 7% CO_2_. After 24 h for the IFN-γ and IL-2 assays, 48 h for the IL-4 assays, and 72 h for the IL-17 assays, (as specified) of incubation, the PBMC were removed, and the detection antibody and development reagents from the kit were added. Following completion of the ELISPOT assay the plates were air dried in a laminar flow hood prior to analysis for the IFN-γ and IL-2 ELISPOT plates were scanned and analyzed using an ImmunoSpot^®^ S6 Ultimate Reader (S6ULT9000, CTL). Spot Forming Units (SFU) were automatically calculated by the ImmunoSpot^®^ Software for each antigen stimulation condition and the medium (negative) control using the SmartCount™ and Autogate™ functions [31].

### 2.4. Cell Separation and Flow Cytometry

CD4 and CD8 T cells were depleted from PBMC by positive selection using EasySep positive selection cocktail containing monoclonal antibodies coupled to Magnetic Nanoparticles (Stemcell Technologies, Vancouver, BC, Canada). After the cell separation total CD4 and CD8 T cell subsets were assessed by flow cytometry. Briefly, 1,000,000 cells were stained in 5 mL Falcon round bottom tubes with human anti-CD4 FITC and CD8 PE (all BD Biosciences, San Jose, CA, USA) and fixed in 2% formaldehyde. A minimum of 200,000 events were acquired using BD FACSCanto III (BD) and analyzed with FACSDivaTM, version 4 software.

## 3. Results and Discussion

### 3.1. Optimizing Test Conditions for Accurate T_H_1, T_H_2, and T_H_17 Frequency Measurements

T cells maintain their antigen receptor specificity over time and, therefore, the frequency of T cells with a given antigen specificity is a result of the expansion of reactive clones and can be measured precisely. This rule also applies to HCMV-specific T_H_1, T_H_2, and T_H_17 cells. When such frequencies are established by a functional assay – in this case by measuring the numbers of antigen-specific T cells that secrete the respective signature cytokines for the T_H_1, T_H_2, and T_H_17 lineages – the assay conditions need to be optimized so that all antigen-specific T cells become activated, and that the analyte secreted is measured at single cell resolution. In preparation for establishing the frequency of HCMV specific T_H_1, T_H_2, and T_H_17 cell in our donor library, we needed to determine (a) the time point of maximal secretion for each cytokine, (b) the distributional properties of the respective cytokine spots for accurate counting, (c) the linearity of measurements, (d) the optimal antigen dose, and (e) that the antigen-triggered cytokine is secreted by antigen-specific CD4 cells as opposed to resulting from bystander reactions.

#### HCMV Grade 2 Antigen-Induced IFN-γ, IL-2, IL-4 and IL-17 ELISPOTs Peak on Different Days after Antigen Stimulation

Antigen-specific T cells are typically in a resting state in the blood (in PBMC), and such cells do not secrete cytokines [32,33]. Activated T cells, that would secrete cytokine, are present in blood only in very low numbers because such T cell blasts are non-recirculating and rapidly home into peripheral tissues [34]. Subsequently, the medium control of ELISPOT assays in which T cells along with the other cell lineages contained in PBMC are cultured in medium alone, in the absence of antigen, show no spontaneous cytokine production, or very low spot counts.

Addition of the test antigen, UV-inactivated virions of HCMV Grade 2 antigen, to the PBMC culture, results in processing of the viral proteins in antigen presenting cells (APC) and the presentation of viral peptides on MHC Class II molecules of the APC for CD4 T cell recognition. If the PBMC contain antigen-specific memory T cells due to a previous immune response, these T cells become activated. Subsequently, the T cells transit from the resting memory cell state (in which they do not produce cytokine) into a T cell blast state, in which they start to secrete the cytokine that they learned to express through instructed differentiation [35]. For measuring T cell-produced IFN-γ it has been established for various antigens that a 24 h stimulation culture is optimal in ELISPOT assays [36]. Measuring cytokine producing cells at the peak of production is critical to establish the accurate frequency of T cells secreting that cytokine. Therefore, one of the primary scopes of this work was to establish whether 24 h stimulation was optimal for HCMV grade 2 to induce IFN-γ secretion of antigen-specific T cells. We also wanted to investigate whether other cytokines of interest were also secreted following the same kinetics.

**Figure 1:**
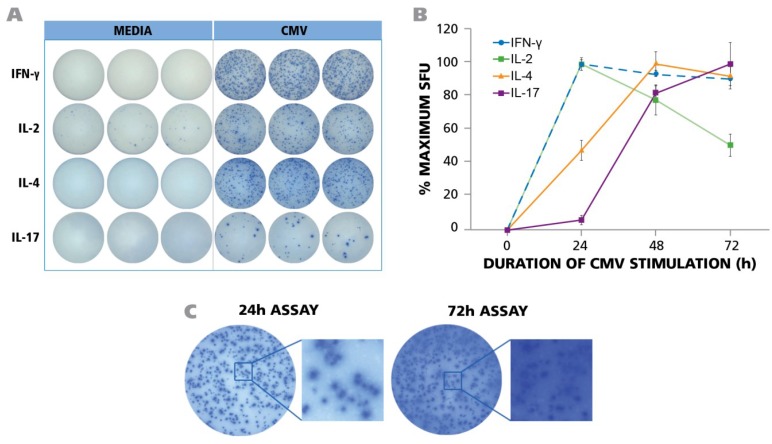
PBMC from 45 donors was tested in ELISPOT assays without adding HCMV (antigen) or in the presence of UV-inactivated HCMV virions. The assay was performed as described in Materials and Methods with 250,000 PBMC per well and 50 μg/mL of HCMV antigen. (**A**) Representative well images of one donor for medium control and antigen-specific IFN-γ, IL-2, IL-4, and IL-17 assays are shown at the time point of peak secretion; (**B**) PBMC was stimulated with HCMV in ELISPOT assays for 24 h, 48 h, and 72 h. IFN-γ, IL-2, IL-4 and IL-17 responses exhibited as spot forming units (SFU) were recorded at each time point. (*n* = 3) (**C**) Maximal number of IFN-γ spots was observed at 24 h. At later time points (72 h) the ELISPOT image was over developed.

In order to establish the secretion kinetics of the signature cytokines of the major CD4 lineages, we measured IFN-γ, IL-2, IL-4, and IL-17 production in the respective ELISPOT assays, 0, 24, 48, and 72 h after addition of HCMV-grade 2 antigen compared to medium control wells only. Less than 10 spots per well were seen in the media control for all cytokines, and at all 4 time points (data are shown in Figure 1A for the optimal time points only). For IFN-γ, 24 h was found to induce the maximal spot counts, and also optimal spot definition. (Figure 1A,B—This data confirms our previously published finding [37]).

At 48 and 72 h, spot counts decreased (Figure 1B) and spots became fuzzier (Figure 1C). This outcome is known to result when cytokine production stops and the plate bound analyte (here IFN-γ) spreads by lateral diffusion [38]. By 72 h this trend continued for IFN-γ (Figure 1C). The IFN-γ spot numbers elicited in different donors underlied a wide variation, ranging from 0 spots to 925 spots per well (see Supplementary Table 1), but for all six donors for whom the IFN-γ kinetics was tested, spot numbers and definition peaked at 24 h. Expectedly the frequency between individual donors varied (see Supplementary Table 1), therefore, in order to account for the variable maximal frequency, the data in Figure 1B are shown as % maximal response. Also the frequencies of HCMV grade 2 antigen-induced IL-2 spots showed a wide range from zero spots to 490 spots (see Table S1) with maximal IL-2 spot numbers detected at 24 h (Figure 1B). The HCMV-induced IL-4 spot counts ranged from 0 to 452 (see Table S1), however, the production of this cytokine showed a delayed kinetics. By 24 h, only about half maximal spot counts were induced, peaking at 48 h, and starting to decline by 72 h (Figure 1B). In contrast, for IL-17, only 6% maximal spot counts were elicited by 24 h, and numbers peaked at 72 h (Figure 1B). Supplementary Table S1 shows the range of IL-17 spots detected at 72 h.

Therefore, the production of T_H_1 signature cytokine IFN-γ, of the T_H_2 cytokine IL-4, and T_H_17 signature cytokine IL-17 showed fundamentally different secretion kinetics. Measuring IL-17 at the time point that is ideal for IFN-γ would lead to a gross underestimation of T_H_17 cell frequencies, and so would measuring IFN-γ at the optimal time point for IL-17. To accommodate for the above differences in cytokine secretion kinetics, we performed the respective cytokine ELISPOT assays for the PBMC library characterization at the respective peaks, 24 h for IFN-γ and IL-2, 48 h for IL-4, and 72 h for IL-17. The raw data for such frequencies are shown in Supplementary Table 1, and will be analyzed below. Representative assay results are shown in Figure 1A. As can be seen, all these cytokine assays provide spots with pristine morphology when tested under optimized conditions.

### 3.2. Log Normal Size Distribution of HCMV-Induced IFN-γ, Il-2, IL-4 and IL-17 Spots Permits Statistics Based Objective Counting

**Figure 2:**
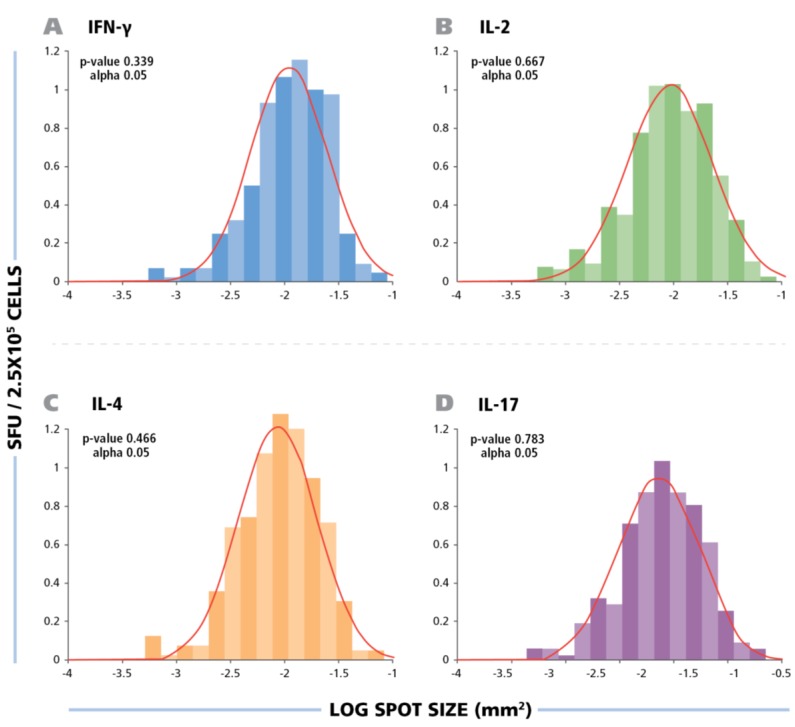
The spot size distribution for different cytokines follow Log Normal distribution. The experimental size distribution of typical recall responses are shown as histograms for the specific cytokines (IFN-g, IL-2, IL-4, and IL-17) with the theoretical Log Normal distribution curve overlaid in red. To test for normality, Kolmogorov-Smirnov goodness of fit was used.

As can be seen in Figure 1A, the spot sizes for all four cytokines showed a wide range. In our previous work we showed that the IFN-γ spots produced by CD8 cells follow Log Normal distribution [39]. This notion was recently verified for both CD8 and CD4 cells using a large number of PBMC donors and involving several antigens [40]. Knowledge of the distributional properties of such spots permits us to apply a statistical approach to define a large spot *vs.* a cluster of spots, and the smallest spot that should be still counted by eliminating debris. In a Log Normal distribution, 3SD (Standard Deviation) demarks with a 95.5% confidence, the upper and lower limit of spot sizes that belong to the distribution in question. In other words, spots larger than 3SD of the mean spot size represent clusters with this high level of confidence. Spots lower than that does not represent secretory activity by the same population of T cells. It has not been established, thus far, whether the Log Normal distribution of IFN-γ spots also apply for complex antigens such as the inactivated HCMV virus, and whether it would also apply to spots generated in IL-2, IL-4 and IL-17 assays induced by this antigen. Figure 2 shows the size distribution of HCMV induced ELISPOTs for all four cytokines.

Statistical analysis by Kolmogorow-Smirnow test of these spot distributions showed that all of them follow a Log Normal distribution. These data suggest that for counting ELISPOTs in all four HCMV induced cytokines, it is suitable to use a statistics based automated gating function (Autogate of the ImmunoSpot^®^ software) to establish accurate spot counts. All spot counts reported here have been established in this way, therefore, avoiding subjectivity, firmly establishing the frequency of antigen-induced cells within the PBMC population tested. The use of competing technologies, such as intracytokine staining, may be used to determine a higher frequency of events, these data are not subject to stringent statistical gating. The lack of objective parametric statistics leads to subjective counts and therefore differences in frequency measurements.

### 3.3. HCMV Grade 2 Antigen-Induced IFN-γ, IL-2, IL-4 and IL-17 ELISPOTs Are Produced by Antigen-Specific CD4 Cells

Short peptides with known MHC-binding properties are well suited for the use as antigens in T cell assays [41]. While several such peptides of HCMV have been defined for MHC class I molecule binding and CD8 cell activation [41] the class II restricted epitopes recognized by CD4 cells are less known [18,42,43,44]. The HCMV virus is a complex antigenic system. It encodes over 200 predicted open reading frames and there are about 30 to 35 viral proteins that compose HCMV virions that are hundreds of amino acids long each, and therefore contain a very high number of potential antigenic determinants, that will be different for each donor as these donors express unique MHC allele combinations [45]. Based on the HLA diversity of the donors and the complexity of the antigen components that constitute HCMV, performing a study like this with peptides would inevitably mean selecting a fraction of potential determinants. Instead, we opted to use the entire inactivated virus as the antigen. Being inactivated, we hypothesized that the virions are not capable of replicating and, thus, will not lead to antigen presentation on HLA-Class I molecules. Instead, the inactivated virus should behave as extracellular proteins generally do: After pinocytosis and lysosomal processing they will end up being presented on HLA Class II molecules, stimulating CD4 cells [46]. If so, the inactivated virus would be ideal for testing the entire virus-specific CD4 cell repertoire, because all the proteins are presented and the respective MHC molecules expressed by the individual test subjects will define which determinants of the virus are displayed to CD4 cells for recognition.

While working with complex organisms, such as entire viruses, one has the advantage of being able to accommodate the complexities of antigen presentation. However glycosylated proteins, (entire viruses) as opposed to linear peptides, can cause pattern recognition signaling leading to cytokine production by the innate immune system. In such cases the antigen triggered cytokine response would result from cells of the innate immune system, and will not reflect on T cell memory. With all this in mind, we needed to establish whether HCMV grade 2 antigen stimulates CD4 cells and if the stimulation was antigen-specific.

**Figure 3:**
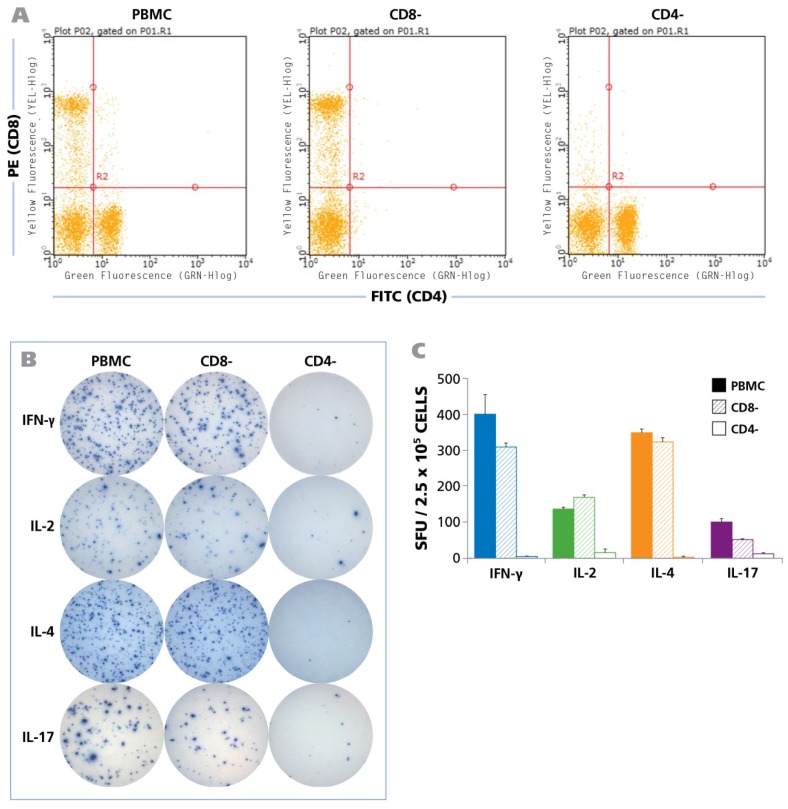
HCMV induced recall response is produced by CD4 cells for all cytokines. Unseparated PBMC, CD8 depleted PBMC, and CD4 depleted PBMC (2.5 × 10^5^ cells each) were tested in human ELISPOT assays to detect the secretion of IFN-γ, IL-2, IL-4, and IL-17 at their appropriate time points as described in Figure 1. CD4 and CD8 depletions were carried out by magnetic bead separations. (**A**) FACS analysis of the unseparated, CD8-depleted and CD4-depleted PBMC populations is shown; (**B**) Representative well images from one donor for the unseparated PBMC, CD8-depleted PBMC, and CD4-depleted PBMC with IFN-γ, IL-2, IL-4, and IL-17 recall responses, following activation with HCMV antigen for 24 h, 24 h, 48 h, and 72 h respectively is shown; (**C**) The mean and SD (Standard deviation) of cytokine spot forming units (SFU) from 2.5 × 10^5^ unseparated PBMC (shown as solid bars), CD8-depleted PBMC (hatched bars), and CD4-depleted PBMC (empty bars) each are shown (n = 6).

**Figure 4:**
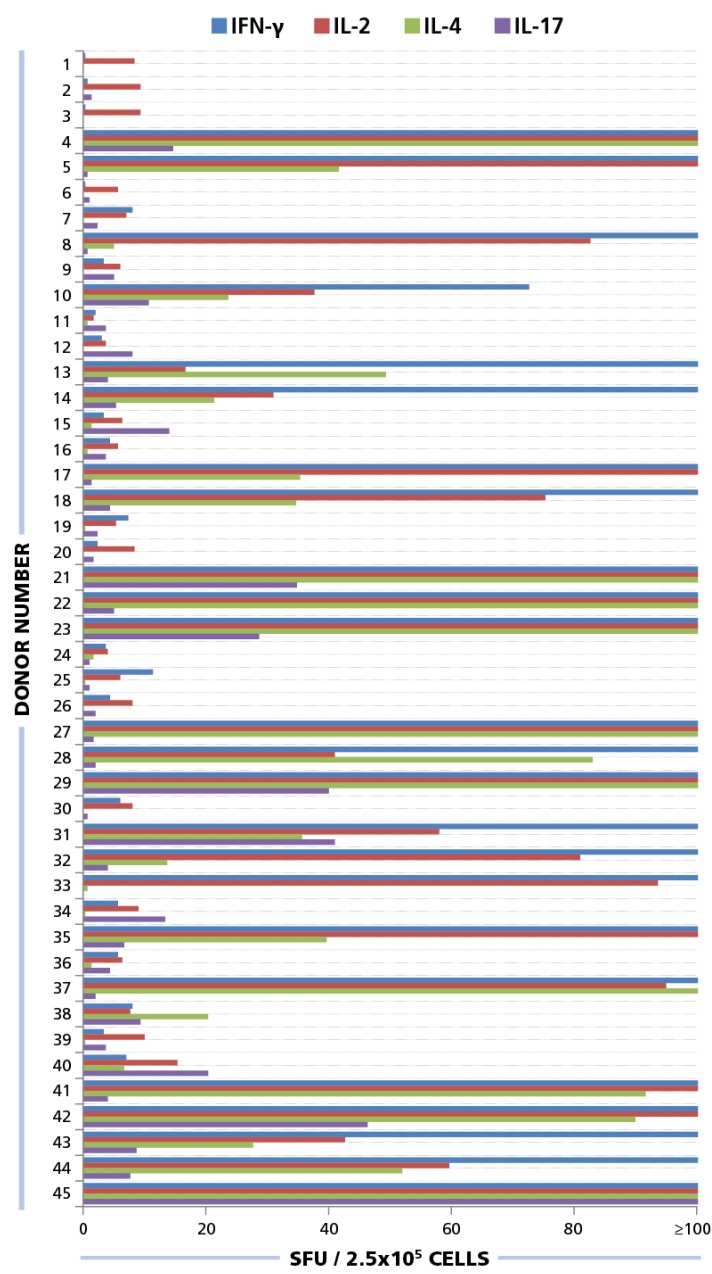
ELISPOT responses for IFN-γ, IL-2, IL-4, and IL-17. PBMC from 45 donors were plated at 250,000 cells/well and 50 μg/mL of HCMV antigen was added to induce a recall response for INF-γ (blue bars), IL-2 (red bars), IL-4 (green bars), and IL-17 (purple bars) from CD4 cells. SFU for each cytokine was established. SFU greater than 100 spots per well are not shown here. Raw data is available as part of Table S1.

To define the cell type that produces the cytokines in question, we tested unpurified PBMC, and PBMC from the same donor from which either CD8 or CD4 cells were depleted by magnetic-bead based selection. The depleted population exhibited no trace of the depleted cell type as can be seen in Figure 3A showing the flow cytometry analysis of CD8-and CD4-stained cells. Testing the three PBMC variants showed that CD8 cell depletion caused a moderate reduction of the spot forming units, while CD4 depletion abrogated it (Figure 3B). Spot counts are shown in Figure 3C. The data show that within PBMC, CD4 cells represent the prevalent cell population that responded with production of IFN-γ, IL-2, IL-4, and IL-17 when stimulated with HCMV Grade 2 antigen. While a moderate decrease was seen in the CD8 depleted cell fraction as well, this is likely to result from the prolonged treatment of the cell sample during the isolation process, rather than from CD8 cells recognizing the HCMV Grade 2 antigen. Also the fact that CD4 cell depletion close to completely abrogated cytokine production argues against a significant participation of CD8 cells, as all CD8 cells are still present in the CD4 depleted fraction. The lack of cytokine response in the CD4 cell depleted PBMC also strongly argues against the possibility of cells of the innate immune system responding to the antigen. Further supporting the latter notion, no cytokines were triggered in PBMC donors that were seronegative to HCMV (Figure 4 and Table S1).

### 3.4. Linearity between PBMC Numbers Plated and Cytokine Spot Counts

T cell activation requires antigen display on a suitable antigen presenting cells (APC) [47]. Thus, at least two cell types are involved in T cell assays such as ELISPOT. In order to detect all antigen specific T cells, one needs to plate the PBMC dense enough so that each antigen-specific T cell can interact with an APC otherwise the T cells would go undetected [48]. We showed for peptide antigen-reactive CD8 cells producing IFN-γ, that the numbers of PBMC plated per well and the spot counts elicited by the peptide follow a linear function between 100,000 and 800,000 PBMC per well [40,48,49,50]. CD4 cells have more stringent activation requirements than CD8 cells. CD4 cells need to encounter less frequent MHC class II positive APC within the PBMC that are capable of processing and presenting the antigen [51]. Therefore, determining whether the PBMC numbers plated and cytokine spots detected for each of the CD4 cell subsets follow a linear correlation is essential to make sure that all antigen-reactive CD4 cells are being registered when the PBMC are tested at a certain cell number per well. Thus, this will also be essential for selecting the right cell number for the assay. Furthermore, if the PBMC numbers and spot count are linear in a certain range, one can conclude that deviations in spot counts are within the precision of the pipetting error.

**Figure 5:**
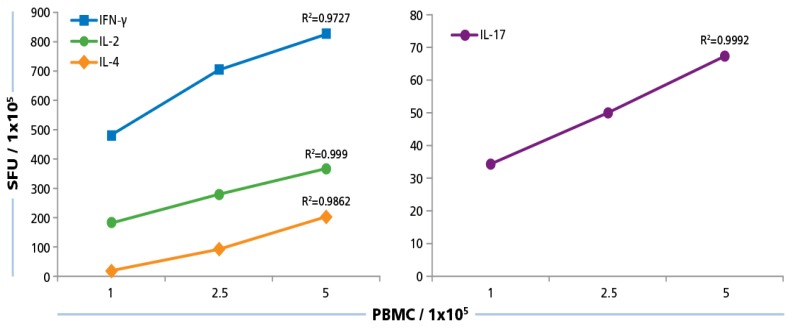
Cell number dependence of ELISPOT formation. PBMC were plated at 100,000 250,000, and 500,000 cells/well and 50 μg/mL of HCMV antigen was added to induce a recall response for INF-γ, IL-2, IL-4, and IL-17 from CD4 cells. SFU at the different PBMC numbers plated were established. Mean values and the regression coefficient (R^2^) from four donors is shown.

We plated PBMC at 100,000, 250,000, and 500,000 cells per well, and tested them with and without HCMV Grade 2 antigen. As can be seen in Figure 5, PBMC numbers and spot counts for all four cytokines were linear with a regression coefficient *R*^2^ > 0.97. Therefore, we selected 250,000 PBMC per well for the subsequent testing of our donor pool, and have established that variations in spot counts resulting from pipetting inaccuracies affect the spot count only by the magnitude of the error itself that follows predictions of a Normal distribution [52].

### 3.5. The Maximal Stimulatory Concentration of HCMV Grade 2 Antigen Is the Same for Eliciting All Four CD4 Subsets

Most T cell responses are polyclonal, that is, the T cells utilize different T cell receptors (TCR) for recognition of the antigen. These TCR can have a spectrum of affinity for the antigen, ranging from low to relatively high that govern repertoire selection processes, including positive and negative selection in the thymus [53], and antigen-driven clonal expansions [54]. The antigen dose response curve reflects the overall avidity of the polyclonal antigen-specific CD4 cell population [55]. This determines the maximum stimulatory antigen dose and the subsequent plateau level at which all antigen-specific T cell clones including low affinity ones become activated. Functional assays that aim at establishing the frequency of all antigen-specific T cells need to be performed at this plateau level. These experiments were performed to establish the maximum stimulatory HCMV dose. Since T_H_1, T_H_, and T_H_17 cells utilize different co-stimulatory and signaling pathways, we needed to establish the dose response curves for all signature cytokines.

**Figure 6:**
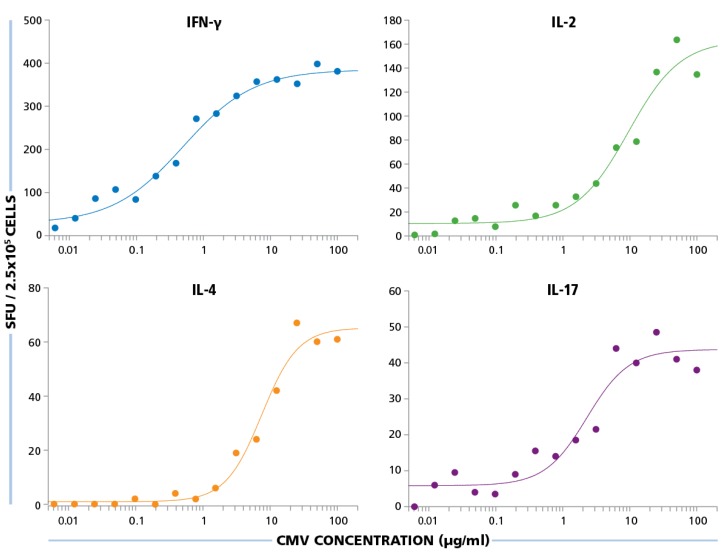
Establishing optimal antigen dose for HCMV-specific CD4 cell stimulation. PBMC were plated at 250,000 cells per well and was stimulated with different concentrations of HCMV antigen starting from 100 μg/mL to 0.01 μg/mL. SFU recorded from IFN-γ, IL-2, IL-4, and IL-17 ELISPOT assays are shown for each of these antigen concentrations.

The HCMV Grade 2 antigen concentration was titrated between 100 μg/mL and 0.01 μg/mL, and PBMC were tested at 250,000 cells per well, after a 24 h assay duration for IFN-γ and IL-2, 48 h for IL-4, and 72 h for IL-17. As shown in Figure 6, for all four cytokines, the dose response curves started to approximate plateau values at 10 μg/mL and safely reached plateau at 50 μg/mL. Therefore, for all subsequent experiments, the HCMV antigen concentration used was 50 μg/mL.

### 3.6. The Frequencies of HCMV-Specific TH1, TH2, and T_H_17 Cells in 45 Healthy Human Donors

The above experiments suggested the following as the optimal test conditions for measuring frequencies of HCMV-specific T_H_1, T_H_2, and T_H_17 cells: (a) Plating PBMC at 250,000 cells per well would be in the linear range; (b) the antigen dose at 50 μg/mL would allow maximal stimulation of the cytokine and (c) the assay duration at 24 h for IFN-γ and IL-2, 48 h for IL-4, and 72 h for IL-17 would result in peak cytokine stimulation followed by (d) counting spots with gating based on Log Normal distribution. Using these parameters, we systematically tested PBMC of 45 healthy donors in ELISPOT assays. The raw data comprising all test results are shown in Table S1.

**Figure 7:**
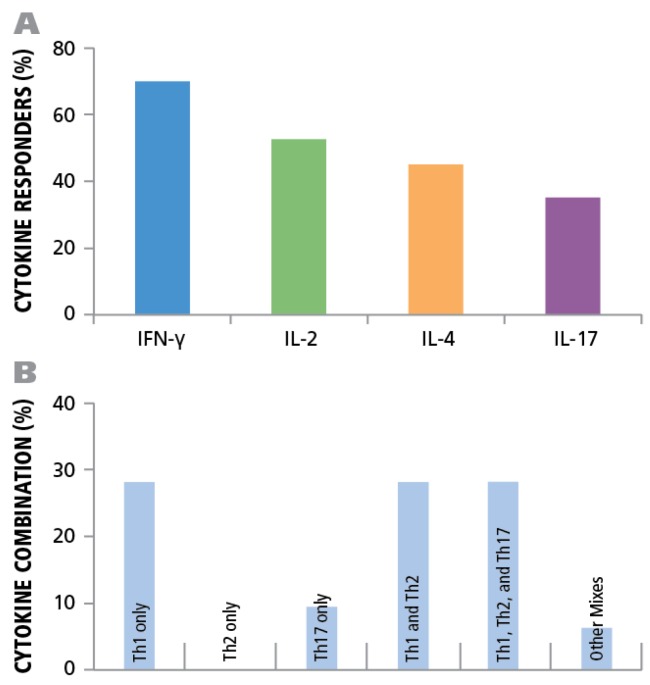
Establishing cytokine signature of CD4 cells. PBMC from 45 healthy donors were tested for HCMV-induced cytokine production of IFN-γ, IL-2, IL-4, and IL-17. (**A**) The percentage of donors that generated a recall response to the HCMV antigen for each of the cytokines is shown. (**B**) The percentage of individual donors representing the various CD4 effector lineages is shown.

Of the donors that exhibited a CD4 response to HCMV grade 2 antigen, 70% displayed an IFN-γ, 52.5% IL-2, 45% IL-4, and 35% IL-17 response by CD4 cells (Figure 7A). The frequencies for each cytokine showed a wide distribution. As can be seen in Supplementary Table 1 high frequency production of one cytokine class, e.g., IFN-γ produced by T_H_1 cells, was not necessarily linked with high frequencies of the other cytokine producing cells. Thus, in the donors tested, there was no consistent ratio for T_H_1, T_H_2, and T_H_7 responsiveness. Rather, the following patterns could be observed (Figure 7B): IFN-γ was seen in most donors (28%) in isolation (9 of 32) or with other cytokine signatures. IL-17 was also seen in isolation (3 of 32) or in combination (Figure 7B). All donors that had positive cytokine recall responses were either positive for IFN-γ or for IL-17, or for both. Therefore, it appears that both T_H_1, and/or T_H_17 immunity can be associated with an HCMV controller status. In contrast, no IL-4 recall responses were seen in isolation, but occurred frequently in combination with IFN-γ (18 of 32) and in two donors with IL-17, but without IFN-γ (Figure 7B). Therefore, T_H_2 immunity does not appear to be suited for controlling the virus, but seems to be consistent with a carrier status, and potentially beneficial, when it occurs together with T_H_1 or T_H_17.

Our data does not determine the absolute frequency of CD4 cells. It only establishes the frequency of T_H_1, T_H_2, and T_H_17 cells. There are new lineages emerging (IL-9 producing T_H_9 cells, IL-21 producing T_H_21 cells) and possibly others that the scientific community is not aware of. As the latter have not been studied, yet constitute part of the total antigen-specific CD4 cell repertoire.

### 3.7. Polyfunctional T Cells Were Present in Most Test Subjects

While IFN-γ, IL-4, and IL-17 are typically expressed in a mutually exclusive manner by the dedicated T_H_1, T_H_2, and T_H_17 CD4 subsets respectively, IL-2 can be co-expressed along with IFN-γ [56,57]. Therefore, when studying co-expression of cytokines we focused on IL-2 and IFN-γ measurements, where co-expression can be expected. The other cytokines were studied individually as co-expression was not expected. IL-2 is also frequently expressed in isolation (without IFN-γ, or IL-4, or IL-17) by a T cell subset called T_H_pp that is thought to represent uncommitted precursor T cells capable of differentiation into T_H_1, T_H_2, or T_H_17 cells [58]. It has been observed in several infectious diseases that the number of T cells that co-express IL-2 and IFN-γ, so called polyfunctional T cells, are positively correlated with protection from the infection [26,59,60,61].

We measured the frequencies of HCMV-specific CD4 cells that secrete either IFN-γ alone, IL-2 alone, or both simultaneously (*i.e.*, are polyfunctional). A double color assay was performed using substrates that produce pure red and pure blue precipitates and that we have previously shown to detect double producers with the same sensitivity for cytokine co-expression as intracytoplasmic cytokine staining [62]. Cytokine spots were seen that were either red (IFN-γ), or blue (IL-2), or of mixed color, purple, and therefore double positive, *i.e.*, polyfunctional. The cells were tested in parallel at two concentrations—150,000 and 300,000 cells per well, to determine whether spots that are double positive reflect the secretion of a single cell or the overlay of two single positive cells. The chance of overlay exponentially decreases when the cells are plated at lower numbers and double-positive cells must occur at the same frequency for both cell densities.

**Figure 8:**
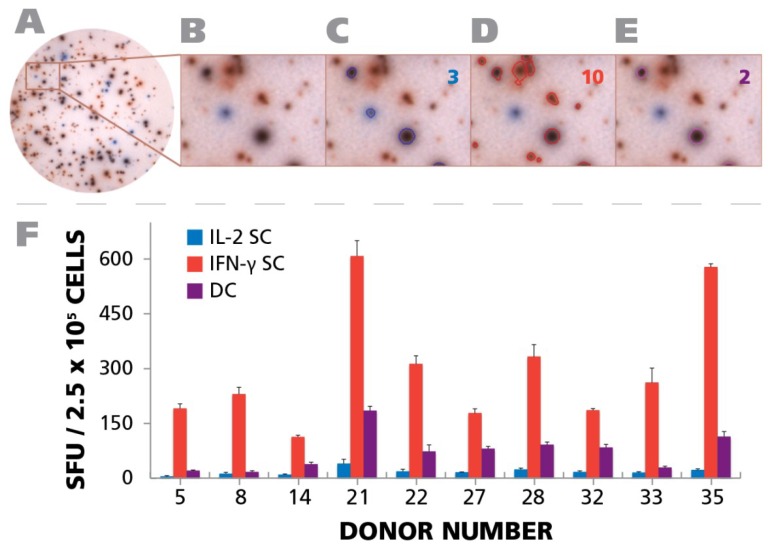
Detection of polyfunctional CD4 cells. PBMC from 10 donors (IDs are specified in the figure) were subject to an IFN-γ/IL-2 Dual color ELISPOT assay at 250,000 cells per well, upon activation with 50 μg/mL of HCMV antigen. PBMC donor IDs were selected based on the response observed in Figure 4A. (**A**) A representative well image with both IFN-γ and IL-2 response is shown; (**B**) A digitally magnified portion of the representative well is shown; (**C**) Blue colored SFU representing IL-2 response are overlaid with a blue circle. The number of IL-2 SFU is inset in the figure; (**D**) Red colored SFU representing IFN-γ response are overlaid with a red circle. The number of IFN-γ response is inset in the figure; (**E**) Responses that are positive for both IL-2 and IFN-γ are shown as SFU that are both blue and red, and are visually represented by a purple color. The number of polyfunctional, *i.e.*, positive for both, responses is inset in the figure; (**F**) The mean recall response for IFN-γ, IL-2, and polyfunctional responses for the individual donors and their respective SD from three representative wells is shown.

A representative well is shown in Figure 8A, and a magnified well segment in Figure 8B. Using the ImmunoSpot^®^ Double Color analysis suite, that was specially developed for double color ELISPOT analysis, we counted the assay results. Figure 8C,D, and E show the outlines of the spots that were recognized in the example provided by the software as pure blue, pure red and double color, respectively, with the spot counts shown in the respective colors in the right corner of the panels. As can be seen in Figure 8F, in all test subjects the majority of cells secreting IL-2 and/or IFN-γ were single positive. In all test subjects double positive cells constituted between 45.4% and 7.4% of the IFN-γ positive cells. The fraction of IL-2 only positive cells was invariably lower than that. Therefore, in the subset of HCMV controllers that exhibited a T_H_1 (IFN-γ) population, polyfunctional T cells were invariably present. However, as shown above (Figure 7B), in 9.4% of the test subjects a T_H_17 response occurred in the absence of IFN-γ and IL-2, thus, such subjects controlled HCMV in the absence of detectable polyfunctional T cells. Overall polyfunctional T cells were present in most test subjects and therefore associated with a controller status but, like T_H_1, did not seem to be essential if T_H_17 immunity is present.

### 3.8. Longitudinal Study of the Frequency of HCMV-Specific T_H_1, T_H_2, and T_H_17 CD4 Cells Shows Relative Stability in Individual Test Subjects

HCMV persists in infected individuals, and reactivates subclinically from time to time, even in healthy subjects [2,3]. Similar to renewed antigen stimulation/boosting in general, one might expect that the reactivation leads to renewed rounds of clonal expansions by the antigen-specific lymphocytes, and possibly also continued T_H_1/T_H_2 or T_H_17 polarization. After unknown rounds of such amplificatory responses, it can be expected that continued antigen encounter leads to exhaustion of the HCMV-specific T cells. The frequency of HCMV reactivation in healthy donors is not clear, and neither is the impact it has on the HCMV-specific CD4 cells [4]. Longitudinal studies may help answer this question.

**Figure 9:**
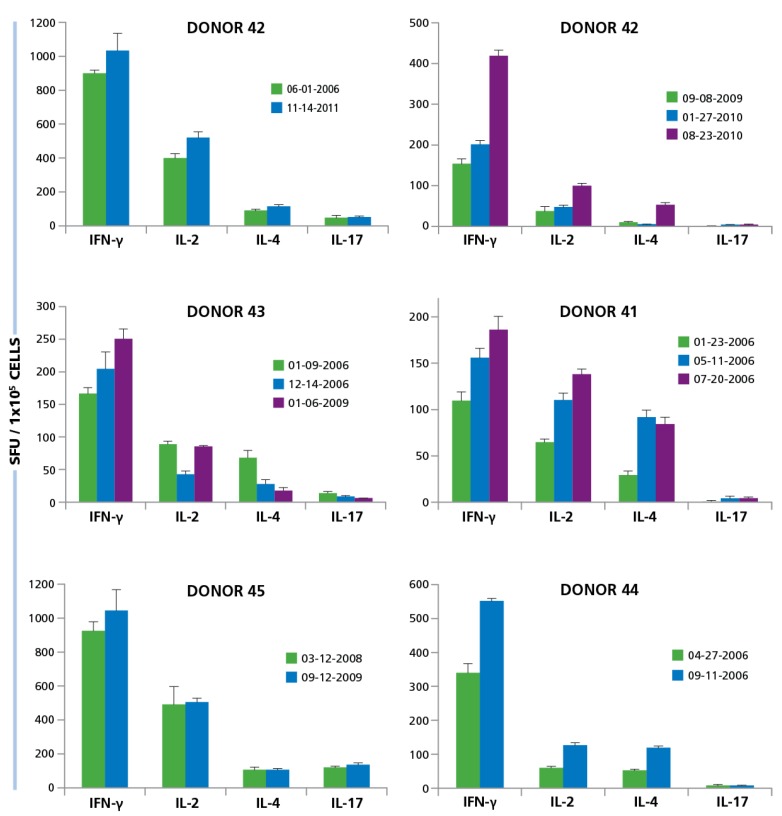
Longitudinal studies of donors show similarity in responses over multiple years. PBMC from six different donors were isolated at different time points over years. Some donors were tested multiple times within 1 year (Donor ID 13, 41, 44), others within 3 years (ID 43, 45) and one donor was tested over 5 years (ID 42). The dates at which PBMC was isolated is specified in the figure for each donor. PBMC isolated from each donor at the different time points, at 250,000 per well, was activated with 50 μg/mL HCMV antigen and the cytokine response for IFN-γ, IL-2, IL-4, and IL-17 was determined. Mean and SD of SFU from three repetitive wells are shown.

For six of our test subjects we could gain repeated access to their PBMC. We gained access to one donor’s (Donor 42) PBMC five years apart. Testing of both isolates showed essentially identical frequencies of HCMV Grade 2 antigen-specific CD4 cell secreting IFN-γ, IL-2, IL-4, and IL-17 (Figure 9). Another test subject (Donor 43) was studied three years apart. Comparing the overall frequencies, we observed that the responses were mostly similar, although slightly increased for IFN-γ, slightly decreased for IL-2 and IL-17, but a marked decrease for IL-4 was seen. When PBMC were collected from the same individual a year later, the increasing tendency for IFN-γ continued, IL-2 returned to the initial level, and the drop in IL-4 and IL-17 stabilized at the level of the bleed two years ago. Four donors were bled two to three times within a year. All showed the tendency for developing increased frequencies of IFN-γ and IL-2 producing HCMV-grade 2 antigen-specific CD4 cells. The ratio of IL-4 and IL-17 producers versus the IFN-γ producing cells did not change substantially but showed moderate fluctuations. In general, when cryopreserved PBMC are tested in the same experiment, as was done with these samples obtained by repeated draws, the experimental error in such experiments is around 20% [49]. Therefore, the changes that exceed such reflect on actual frequency changes *in vivo*. The data showed that there are such fluctuations, but they are not major within an observation period of several years and, while they show substantial inter test subject variations, for a given test subject, the ratio of T_H_1, T_H_2, and T_H_17 cells stay rather constant.

### 3.9. IL-7 Restores Functionality of Exhausted HCMV-Specific CD4 Cells

CD8 cells can undergo exhaustion when antigen persists [9,63,64]. Exhausted T cells lose their ability to secrete IFN-γ and IL-2, but can regain such when exposed to IL-7 [10,65,66]. Presently, it is not known whether the CD4 subsets T_H_1, T_H_2, and T_H_17 cell can exhaust, too, and whether they are also amenable to reactivation by IL-7. Due to the persistence of HCMV in infected individuals, we addressed this question as well.

**Figure 10:**
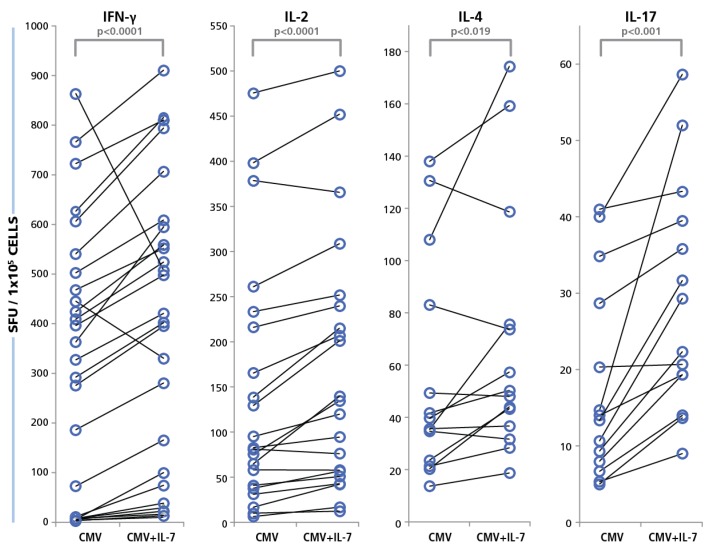
Restoration of functionality of exhausted CD4 cells with IL-7. PBMC from 40 donors were tested in an ELISPOT assay with 50 μg/mL of HCMV antigen, with and without IL-7 added to the culture. Cytokine recall response for IFN-γ, IL-2, IL-4, and IL-17 was recorded as SFU. PBMC were plated at 250,000 cells and the concentration of IL-7 was 30 ng/mL. The SFU with and without IL-7 for each of the cytokines is shown.

The PBMC were tested in the presence or absence of IL-7. The concentration of IL-7 was 30 ng/mL that we established as maximally stimulatory (data not shown) confirming other studies [65]. As shown in Figure 10, increased HCMV grade 2 antigen-specific spot counts were seen for most test subjects in the presence of IL-7, and for all four cytokines. The medium background for all four cytokines was unaffected for all cryopreserved PBMC tested (data not shown). The IL-7 effect was most pronounced for IFN-γ and IL-17, where a maximum increase of 16-fold in HCMV-induced spot counts was recorded. The average increase for IL-17 spot numbers were approximately two-fold and the average increase in IFN-γ spot numbers were about 2.8-fold. On comparing this data with the Non parametric Wilcoxon signed-rank test, the responses of individual donors with and without IL-7 exhibited significant increases for all cytokines (Figure 9). These data suggest that about half of the HCMV-specific CD4 cells in circulation in blood have undergone exhaustion that can be overcome in the presence of IL-7.

We also tested the response of CD8 cells using the same concentration of IL-7 as described above, and stimulating PBMC with a CEF (Cytomegalo-, Epstein Barr-, and Flu- virus) peptide pool, or CEF-7 (a peptide of the HCMV pp65 protein). The number of peptide antigen-induced spot forming units between the PBMC stimulated with and without IL-7 did not exhibit any striking differences and did not show any statistical differences. The data is presented in Figure S1.

## 4. Conclusions

The data communicated here provide, to our knowledge, the first systematic high resolution characterization of CD4 cell immunity to HCMV. Since healthy individuals that can effectively control the infection were tested, these data provide insights into the type of CD4 cell responses that are compatible with a controller status. The data suggest that T_H_1 or T_H_17 subsets in isolation or in combination are required for protection. T_H_2 immunity occurred only in combination with T_H_1 or T_H_17, which further implies that T_H_2, in isolation, does not seem to be compatible with protection. Furthermore, IFN-γ/IL-2 co-expressing polyfunctional cells are associated with, but do not seem necessary for, protection. About half of the HCMV-specific T_H_1, T_H_2, and T_H_17 cells are in a state of exhaustion that can be reverted by IL-7. In spite of antigen persistence the frequencies of T_H_1, T_H_2, and T_H_17 cells remain remarkably constant, in individual subjects, over several years.

## Supplementary Files

Supplementary File 1
