# Supplementary material for: Characterization of the HCMV-Specific CD4 T Cell Responses that Are Associated with Protective Immunity
Source: Viruses. 2015 Aug 6;7(8):4414–37. doi: 10.3390/v7082828 (PMC4576189; doi:10.3390/v7082828)
Supplement: Supplementary File 1 [file viruses-07-02828-s001.pdf]

Supplementary Materials

Table S1. Individual responses for IFN-γ, IL-2, IL-4, and IL-17 responses from 45 donors.

Donor	IFN-Y		IL-2		IL-4		IL-17		OD
1	0	±0	8	±1	0	±0	0	±0	0
2	1	±0	9	±3	0	±0	1	±0	0
3	0	±0	9	±1	0	±0	0	±0	0
4	607	±4	398	±12	264	±38	15	±2	0
5	503	±17	216	±14	42	±1	1	±1	2
6	0	±0	6	±1	0	±0	1	±1	0
7	8	±4	7	±2	0	±0	2	±3	0
8	276	±4	83	±9	5	±4	1	±0	3
9	3	±2	6	±2	0	±0	5	±2	0
10	73	±14	38	±8	24	±4	11	±3	2
11	2	±2	2	±1	1	±1	4	±2	0
12	3	±2	4	±2	0	±0	8	±2	0
13	409	±19	17	±4	49	±11	4	±1	2
14	186	±2	31	±2	21	±4	5	±1	3
15	3	±1	6	±1	1	±1	14	±4	0
16	4	±2	6	±3	1	±0	4	±2	0
17	468	±18	165	±19	35	±4	1	±2	2
18	541	±11	75	±5	35	±9	4	±2	3
19	7	±2	5	±2	0	±0	2	±1	0
20	2	±0	8	±3	0	±0	2	±1	0
21	766	±17	476	±29	452	±37	35	±6	1
22	627	±30	379	±8	303	±53	5	±2	3
23	723	±5	233	±4	350	±21	29	±4	3
24	4	±2	4	±3	2	±2	1	±0	0
25	11	±3	6	±2	0	±0	1	±1	0
26	4	±3	8	±4	0	±0	2	±1	0
27	424	±11	129	±1	138	±22	2	±0	3
28	363	±11	41	±9	83	±6	2	±2	2
29	864	±54	261	±20	131	±8	40	±5	2
30	6	±2	8	±1	0	±0	1	±1	0
31	327	±28	58	±6	36	±1	41	±4	3
32	292	±39	81	±8	14	±2	4	±1	3
33	475	±26	94	±2	1	±0	0	±0	3
34	6	±1	9	±4	0	±0	13	±4	0
35	385	±7	139	±23	40	±6	7	±1	2
36	6	±3	6	±1	1	±0	4	±1	0
37	445	±5	95	±20	108	±19	2	±1	3
38	8	±1	8	±3	20	±1	9	±2	2
39	3	±2	10	± 0	0	±0	4	±2	0
40	7	±5	15	±3	7	±7	20	±5	0

Table S1. *Cont.*

Donor	IFN-Y		IL-2		IL-4		IL-17		OD
41	156	±10	110	±8	92	±8	4	±2	1
42	899	±18	399	±27	90	±8	46	±16	2
43	204	±26	43	±5	28	±7	9	±1	2
44	340	±27	60	±5	52	±4	8	±3	1
45	925	±15	490	±106	105	±17	119	±9	3

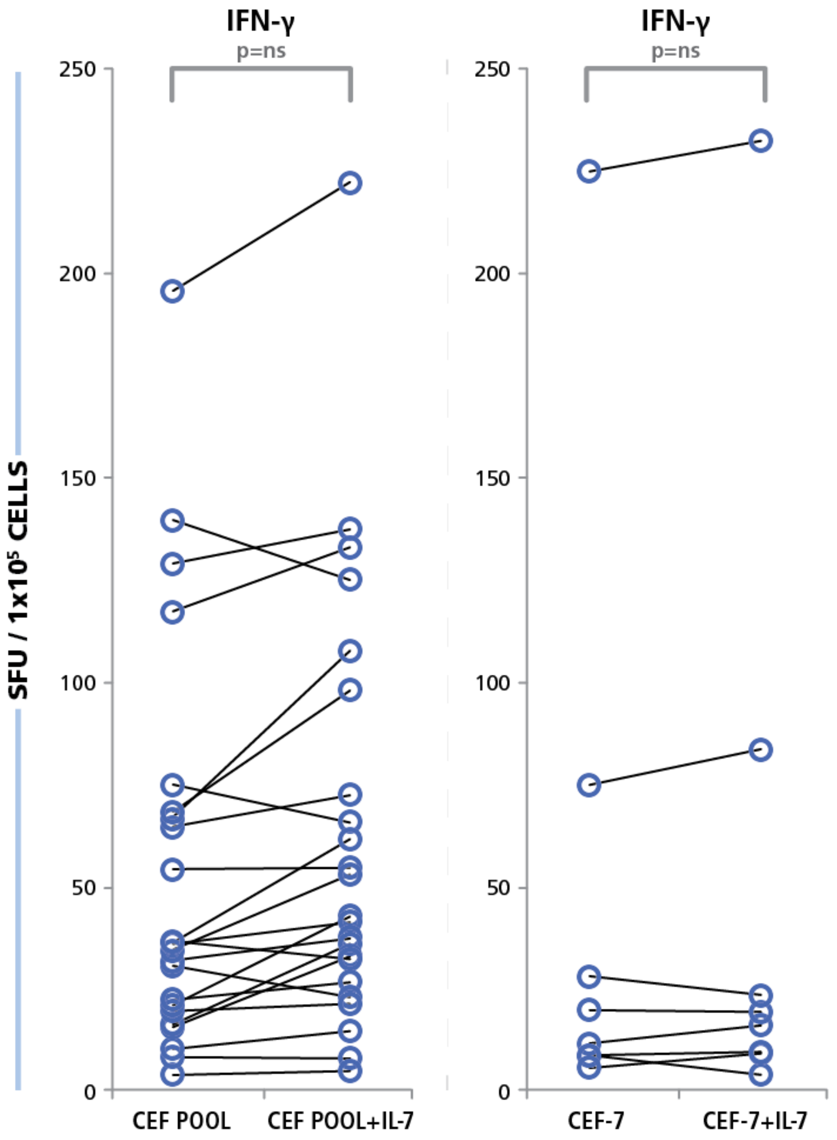

Figure S1. No significant difference in functionality of CD8 cells with IL-7. PBMC from 40 donors were tested in an ELISPOT assay for reactivity to CEF pool, or CEF-7 antigen at 2µg/mL, with and without IL-7 added to the culture. The IFN-g recall response was studied in 250,000 PBMC per well. The concentration of IL-7 was 30 ng/mL. The SFU with and without IL-7 for IFN-γ is shown. Only the positive responses are shown here.
